# PSYCHOMETRIC APPRAISAL OF THE GERIATRIC SOCIAL WORK COMPETENCY SCALE II WITH LIFELONG LEADERSHIP SKILLS

**DOI:** 10.1093/geroni/igz038.1858

**Published:** 2019-11-08

**Authors:** Scott E Wilks, Sarah Choate, Danielle Eugene, Xi Du

**Affiliations:** Louisiana State University, Baton Rouge, Louisiana, United States

## Abstract

The Council on Social Work Education (CSWE) Gero-Ed Center and Hartford Partnership Program in Aging Education (HPPAE) emphasize five competency areas specific to social work practice with older adult clientele (i.e., gero social work), namely knowledge and skills applicable to (a) values, ethics and theories; (b) assessment; (c) intervention; (d) aging programs, services and policies; and (e) leadership in aging environments. Accordingly, CSWE/HPPAE created a standardized measure – Geriatric Social Work Competency Scale II with Life-long Leadership Skills (GSWCS II) – to assess empirically these practice competencies among social work students and gero social work practitioners. A scant amount of literature exists that reports properties of this measure. Consequently, the purpose of this study was to conduct a psychometric examination of the GSWCS II, namely its factor structure and reliability among the competency areas. The sample consisted of three, advanced year MSW cohorts (N=170) from a state flagship university in the southern United States. Almost one-third of the sample were enrolled in a gerontology specialization during their advanced year. The typical participant was a 27-year-old female enrolled full time, completing the 60-credit hour MSW program. Principal axis factor results indicated unidimensionality, using the traditional 1.0 eigenvalue threshold, for each competency scale. All items loaded moderately-to-strongly on their respective competency scales; loadings ranged from 0.569 to 0.906. Regarding internal consistency for each of the competency scales, Cronbach’s alphas ranged from 0.932 to 0.959; Guttman split-half coefficients (lambda-4) ranged from 0.896 to .941. Implications for gero practice competency assessment are discussed.

